# Building CAR-E: A Novel Artificial Intelligence Agent for Coaching Conversations

**DOI:** 10.5334/pme.2563

**Published:** 2026-06-24

**Authors:** Matthew E. Kelleher, C. Y. Zhou, Seth Overla, Andrew Zahn, Brandon Zaffuto, Sally A. Santen, Benjamin Kinnear, Laurah Turner

**Affiliations:** 1 Department of Pediatrics and Internal Medicine, Cincinnati Children’s Hospital Medical Center and University of Cincinnati College of Medicine, Cincinnati, Ohio, US; 2 Division of Pulmonary and Critical Care, University of Cincinnati College of Medicine, Cincinnati, Ohio, US; 3 Office of Medical Education, University of Cincinnati College of Medicine, Cincinnati, Ohio, US; 4 University of Cincinnati College of Medicine, Cincinnati, Ohio, US; 5 Emergency Medicine and Medical Education, University of Cincinnati, Cincinnati, Ohio, US; 6 Department of Medical Education, and Biostatistics, Health Informatics and Data Sciences, University of Cincinnati College of Medicine, Cincinnati, Ohio, US

## Abstract

**Background and Need for Innovation:**

Coaching has many benefits in medical education, but large-scale implementation is constrained by faculty bandwidth, scheduling, cost, and power differentials. Artificial intelligence (AI), specifically large language models (LLMs), offers potential solutions to augment coaching, but limitations remain and refining performance is necessary to create valuable coaching conversations.

**Steps taken for Development and Implementation of Innovation:**

The authors created Coaching with AI-Reinforced Education (CAR-E), an AI coaching agent built using an LLM. Using layered architecture, CAR-E couples real-time speech input and output, retrieval-augmented generation (RAG) for evidence-based coaching, and dual memory system (short-term context and long-term history) to sustain longitudinal dialogue. An iterative design refined CAR-E with users who voluntarily engaged in coaching conversations and provided feedback. Transcripts and user feedback were analyzed to improve CAR-E.

**Evaluation of Innovation:**

During the pilot, 37 medical trainees and faculty engaged in coaching conversations. Transcripts showed diverse topics discussed and many strengths and opportunities for improvement. Users reported that CAR-E facilitated self-reflection, clarified goals, and broke complex problems into actionable steps. They also noted formulaic questioning that felt repetitive, superficial attempts to display empathy and difficulty moving conversations forward. Many users were frustrated by CAR-E’s strict adherence to coaching competencies, which prevented it from giving advice or suggestions.

**Critical Reflection on your process:**

Unlike generic LLMs that default to broad, solution-oriented dialogue, CAR-E was designed for coaching in medical education. Its design incorporates evidence-based knowledge retrieval with curated coaching resources, structured memory to support longitudinal conversation, institutional oversight, and speech integration. These features exemplify responsible AI use in medical education. Despite this architecture, many improvements are planned to create reflective, growth-oriented conversations that augment coaching in medical education.

## Background and Need for Innovation

Coaching is increasingly being used to help physicians and medical trainees manage the demands of training while developing the knowledge, skills, and attitudes necessary to grow and thrive as physicians. Coaching differs from more traditional approaches such as mentoring in that it emphasizes facilitated reflection rather than direct advice or sponsorship. In comparison to mentorship, which provides recommendations based on experience, coaching supports insight, self-awareness, and goal-setting through structured questioning and learner-driven exploration [1,2]. These features are central to coaching conversations and foundational to its integration into medical education. Unfortunately, many obstacles exist to implementing large-scale coaching programs, including available resources, managing schedules, and limited coach training and expertise [3]. In addition, the trainee-coach relationship often involves inherent imbalances in position, power, or identity which might complicate learner trust and comfort with vulnerability [4]. Many of these barriers are less critical to mentoring, but they limit the implementation, and subsequently the impact, of coaching programs within medical education.

Recent advances in artificial intelligence (AI), particularly large language models (LLMs), present unique opportunities to complement human coaching [5]. Coaching depends on human connection and relational trust, whereas *coaching conversations* emphasize the structured process of reflective questioning to facilitate coachee led insights and goal setting within a single interaction. The coaching relationship creates the conditions under which coaching conversations are most powerful, but conversations can generate meaningful reflection even outside an established longitudinal partnership. The latter task affords an opportunity to leverage AI to engage users in meaningful, goal-oriented dialogue without replacing the human relationship central to coaching [6]. However, base versions of commercially available LLMs are not well designed for coaching conversations. For example, LLMs tend to be verbose, provide answers, give advice, and take a sycophantic tone. These characteristics undermine best practices in coaching where users gain insights through reflective question asking, rather than advice giving.

The primary goal of this project was to design an AI agent capable of facilitating coaching conversations that would adhere to coaching competencies and have potential to foster trainees’ personal and professional development [7]. This kind of complex task requires building an AI agent that can perform tasks autonomously, make decisions, and interact with its user to achieve specific goals [8]. In this early phase, we operationalized working as 1) fidelity to coaching principles 2) functional conversational performance and 3) positive user experience. We detail our targeted approach in developing CAR-E (Coaching with AI-Reinforced Education), an innovative AI coaching agent that leverages several technologies to engage in coaching conversations.

## Steps Taken for Development and Implementation of Innovation

**Figure 1:**
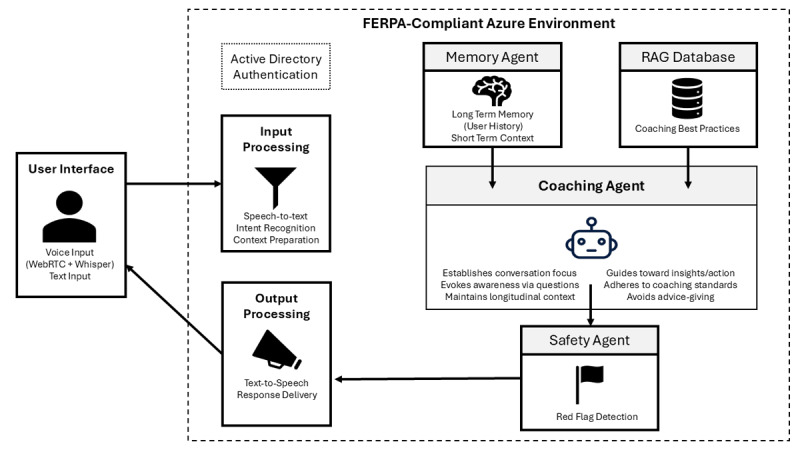
CAR-E system architecture showing the flow from user input through processing, coaching generation, and safety monitoring within a FERPA-compliant environment. The coaching agent (GPT-4) integrates retrieval-augmented generation (RAG) for evidence-based coaching methods and dual memory systems for longitudinal conversation continuity. A safety inference layer screens all outputs before delivery. Authentication via institutional Active Directory ensures appropriate governance and privacy protection.

CAR-E integrates several technologies into one cohesive system (Figure 1) detailed below along with our rationale for why the design principle is important to our overall goal.

1. **Large Language Model (LLM) Foundation:** generating responses that ask open-ended, purposeful questions while limiting information delivery that can undermine the learner’s opportunity for self-discovery.
2. **Voice Interface:** Text-to-speech (TTS) and speech-to-text (STT) since coaching is inherently conversational and relational, speaking and listening allows for better dialogue.
3. **Knowledge Integration:** Retrieval-augmented generation (RAG) to anchor the system in established coaching frameworks and competencies to maintain consistency and reinforce best practices in coaching.
4. **Memory Architecture:** Short-term and long-term memory to enable continuity across conversations. Our priority was short-term to ensure conversations maintain context and coherence, allowing CAR-E to draw upon earlier insights and revisit the users’ values and goals.
5. **User Experience:** A minimalist, accessible interface that lowers the barrier to engagement, encourages repeated use, and ideally keeps the focus on reflection and awareness.

### Technical Architecture Design Principles

The backbone of CAR-E is an LLM. At its core, each user input is processed by the LLM as a prompt, which then returns a response according to prior embedded instructions. Prompts automatically include the full conversation history, allowing the LLM to maintain context.

Basic LLM applications rely on text, which is not ideal for coaching conversations. CAR-E incorporates technologies that enable real-time audio/visual communication and provides accurate transcription. This combination allows users to speak directly with the coaching agent and receive real-time responses, simulating human conversation.

CAR-E uses retrieval-augmented generation (RAG) to access curated coaching materials. Documents were compiled or created by the development team to summarize best practices for coaching in medical education [9]. The backbone of these were the International Coaching Federation (ICF) Core Competency model to serve as CAR-E’s evidence-based design. These training materials “remind” the LLM of coaching principles to avoid defaulting to advice-giving.

CAR-E features memory functionality. “Short-term memory” stores conversational context by including prior dialogue in each new API call, thereby allowing the model to “remember” prior context and maintain coherence. After a session, the conversation is summarized and saved in a long-term memory database tied to the user profile via active directory integration. This architecture enables CAR-E to reference past interactions, track goals, and sustain a longitudinal coaching relationship. The front-end interface is built for users to access CAR-E via a webpage featuring a minimalistic chat window, a menu of conversation topics, and tools for notetaking, goal setting, and reviewing prior conversations.

Given the importance of governance and data security, the platform was developed and hosted within our institution’s secure Microsoft Azure environment under a HIPAA- and FERPA-compliant agreement with Microsoft that includes a Business Associate Agreement. Artificial intelligence processing was performed using Azure OpenAI Service rather than the public OpenAI API. Under this configuration, prompts and generated outputs are not stored by OpenAI and are not used to train external models; all data remain within the institutional Azure environment. The system architecture separates identity management from AI processing. Students authenticate through institutional single sign-on, but no personally identifiable information (e.g., names, email addresses, or student identification numbers) is transmitted to the AI model. These considerations are particularly important given the sensitive and potentially vulnerable nature of coaching conversations.

### Iterative Testing

The development of CAR-E relied on an iterative human-centered design methodology. Human-centered design recognizes that successful technology must align with human cognitive patterns, communication preferences, and workflow requirements to achieve meaningful adoption and impact [10]. Our approach was thus grounded in understanding real user contexts, observing actual usage patterns, and incorporating user feedback to create a more intuitive and effective platform. While all eight ICF competencies informed the original design and RAG corpus, iterative improvements placed particular emphasis on the three that most directly relate to coaching conversations. Competency 6 (*Listens actively*) involved CAR-E’s ability to reflect and summarize communication, notice trends in emotion, and recognize when there is more beneath the surface. Competency 7 (*Evokes awareness*) related to CAR-E’s use of questions that facilitate insight and help the user explore beyond their current thinking. Competency 8 (*Facilitates client growth*) was part of CAR-E’s ability to move the user forward and turn insight into action [11].

Test users for the initial iterative cycles consisted of few faculty and trainees on the core team. Multiple issues were noted with the initial version, often related to conversational flow or technical issues (Table 1). For example, some noted that CAR-E’s responses were exceedingly verbose, asked multiple questions at once, or interrupted the user at any pause in speaking. Technical glitches with transcription features or sound were also noted. We made design choices to operationalize the ICF competencies with specific examples such as explicitly prohibiting advice-giving, directing CAR-E to respond with open-ended questions, limited CAR-E to a single question per response and eventually a three-phase conversational framework to encourage forward momentum. As improvements to CAR-E were made, we returned to the core team for feedback (Table 1).

**Table 1:** CAR-E Iterative Design and Refinement Log. The development of CAR-E followed an iterative, human-centered design process. Early cycles were rapid and informal, conducted with the core team and small groups of faculty and trainees; later cycles incorporated larger feedback bursts from volunteer users across the institution. The log below summarizes each cycle for transparency and transferability and reflects descriptive observation rather than formal qualitative analysis.


CYCLE	WHO TESTED	DOMINANT ISSUE OBSERVED	CHANGE IMPLEMENTED	EVIDENCE THAT SUGGESTED IMPROVEMENT

**1**	One faculty member and the core design team	Foundational build and first internal testing: interface and layout, voice features, memory function, technical glitches, and the need to ground responses in coaching methodology rather than generic LLM behavior.	Built the minimalist chat interface with a conversation-topic menu and goal-setting tools; implemented the dual short and long-term memory architecture; assembled a retrieval-augmented generation (RAG) knowledge base of coaching resources to ground responses in evidence-based coaching practice and curb default advice-giving; resolved speech-to-text transcription and audio-playback glitches.	Core team confirmed reliable navigation, transcription, and session-to-session memory recall, and that responses reflected a reflective, coaching-oriented style rather than generic advice-giving.

**2**	Two faculty and one resident	Responses too verbose; multiple questions asked at once; CAR-E interrupted users at natural speech pauses; tone not conversational enough	Revised the system prompt to shorten responses and limit each turn to a single focused question; raised the voice model’s silence threshold so CAR-E waits through natural pauses before replying; tuned tone toward a more conversational style.	Subsequent transcripts showed shorter, single question turns; testers reported noticeably fewer interruptions and a more natural exchange.

**3**	Four faculty and one resident	Conversations lacked a defined focus; no prompting to shift towards setting goals	Added a session-opening step that prompts users to identify the purpose of the conversation; surfaced goal-setting prompts and tools.	Testers more consistently articulated a focus in the session; transcripts showed CAR-E better at prompting user to consider specific goals.

**4**	Feedback burst — 37 faculty, residents, and students (volunteer convenience sample; pilot cohort summarized in Table 1)	Inadequate recognition of and response to red-flag disclosures; formulaic reflect/question pattern; user frustration when CAR-E resisted offering advice	Began building a safety inference layer to detect concerning disclosures (e.g., unhealthy coping) and trigger escalation pathways; began varying the affirmation–question rhythm to reduce formulaic pattern; scoped a hybrid approach permitting occasional acknowledgments, reflections or suggestions when users are stuck or explicitly request input.	Two authors consolidated the 37-user free-text feedback into strengths and areas for improvement (Table 1); these items directly defined the changes tested in further cycles.

**5**	Two faculty and one resident	Weak conversational arc without moving user forward; assumptions about user meaning or significance; insufficient reflections and acknowledgments before advancing	Began implementing a three-phase conversational arc (establishing focus, evoking awareness, and moving toward insight and action) drawn from International Coaching Federation competencies; refined CAR-E to acknowledge and reflect back users statements and remain open-ended without assumptions.	Returning testers observed a clearer beginning–middle–end structure and that CAR-E validated what was said before advancing; informed the more recent comparison conversation shown in Figure 2.

**6**	Feedback burst — 28 faculty, residents, and students	Shallow emotional validation; limited contextual understanding; absence of concrete recommendations; rarely challenges the user	Ongoing improvements to prompt CAR-E to name and reflect underlying emotions before questioning; strengthening use of long-term memory for contextual continuity; piloting the hybrid approach in which CAR-E offers a concrete observation or soft suggestions when a user appears stuck or explicitly ask.	Early results suggest empathy felt less formulaic and that input or suggestions were available on request.


Cycles 1–3 and 5 were small, rapid design cycles with the core team and returning testers; Cycles 4 and 6 were larger feedback bursts with volunteer users recruited by email invitation. “Evidence” entries are descriptive observations drawn from transcript review and free-text user feedback, consistent with the early-stage, non-formal nature of this innovation report.

**Figure 2:**
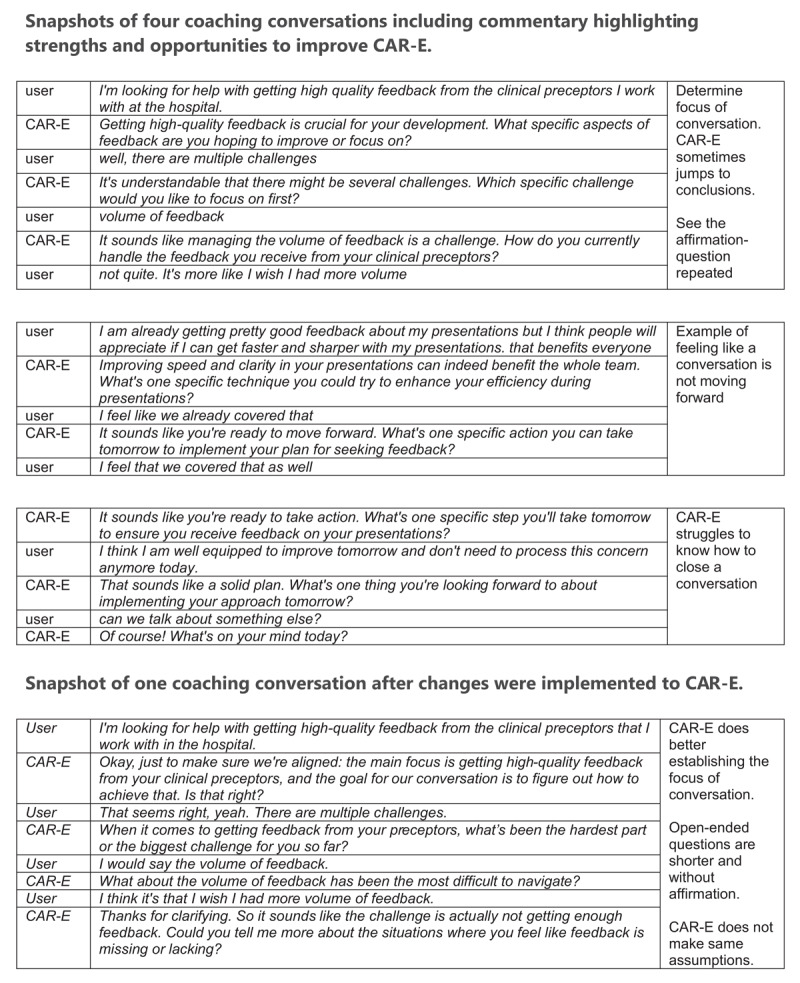
Example coaching conversations illustrating CAR-E behavior before and after revisions, with annotations highlighting common issues. Panel 4 shows the revised CAR-E responding to a similar conversation (feedback volume) after the changes.

After multiple small design cycles, CAR-E was then sent to new users for testing. An email invitation was sent to a convenience sample of 55 students, residents, fellows, faculty, and administrators around the institution to trial CAR-E and leave free text feedback in Microsoft forms. Users were asked to have an honest conversation about something really happening in their lives. Thirty-seven participants engaged with CAR-E and submitted feedback on their experience. Two authors reviewed all responses and grouped comments into features that were working well and areas for improvement—through discussion and consensus (Table 2). This burst of feedback is informing the next iteration of CAR-E and shared here as early, descriptive observations rather than the product of formal qualitative analysis to assess CAR-E’s ability to adhere to specific competences. The University of Cincinnati IRB approved this work as exempt and prior to deployment, the platform underwent institutional IT governance review and independent security evaluation to ensure compliance with institutional data protection standards.

**Table 2:** Organized summary of strengths and opportunities for improvement from user feedback.


STRENGTHS	OPPORTUNITIES FOR IMPROVEMENT

Promoted self-reflection and introspection	Repetitive and Circular Questioning

Questioning was effective	Lack of direct or practical advice

Relentlessly positive in validating concerns	Lack of empathy or human-like interaction

Interaction felt personalized and adaptive	Conversations stagnated without new insight or clear closure

Useful for planning and goal setting	No recognition of red flag responses


## Evaluation of Innovation

Users were encouraged to have a realistic conversation on any relevant topic. De-identified transcripts revealed a wide range of topics discussed. These are organized and included in the appendix. Several topics were repeated and some users engaged in multiple distinct conversations. The feedback from users revealed several insights and are summarized below and in Table 2.

Figure 2 includes four snapshots from coaching conversations in the pilot that illustrate how CAR-E interacts with users. Each snapshot also includes some commentary to highlight strengths and opportunities for improvement. To illustrate the iterative refinement of CAR-E, we have begun making improvements based on the feedback in this innovation report and this figure also includes a more recent coaching conversation on a similar topic.

### User Feedback: Strengths

Users appreciated CAR-E’s ability to facilitate meaningful self-reflection, break down complex problems, and guide them towards actionable next steps. Several were surprised by how well it prompted introspection and helped facilitate their own personalized solutions. Others noted the coach’s skill in validating concerns, identifying underlying emotions and maintaining conversational continuity. Even initial skepticism from some was followed by appreciation for the nuanced questioning that helped to reframe issues and provide greater clarity. Overall, users found the AI coach supportive, thought-provoking, and helpful in fostering insight and problem-solving. This informal review of the feedback suggests that CAR-E is reasonably adhering to ICF competencies 7 and 8.

### User Feedback: Areas for Improvement

Despite its strengths, users identified several limitations. Many felt the conversations could feel formulaic or emotionally sterile, especially during sensitive discussions. Some thought the conversational structure felt repetitive and predictable, noting the “affirmation-question” rhythm that it was trained to follow. For instance, if a user responded with “I’m not sure”, the AI coach tended to rephrase the question rather than shifting to a new line of questioning, suggesting it was following a formula rather than an actual conversation. Other feedback noted that CAR-E responded so rapidly that it made attempts to empathize or validate their concern feel shallow or inauthentic.

Another common insight shared was users struggling with goal setting or ending conversations, a central part of ICF competency 8. Feedback suggested that the AI would keep asking questions until the user ended the conversation or prompted CAR-E to think about action steps. This led many to question if they were actually moving forward in their awareness and understanding of an issue.

One concern was raised when a user stress tested CAR-E with red flag scenarios or coping strategies (i.e. binge drinking, quitting goals, isolating themselves) and felt its recognition and response was inadequate. In one conversation they described escalating alcohol consumption as a coping mechanism and CAR-E appropriately suggested reaching out to a support group or mental health professional. Unfortunately, when more direct advice was requested by the user, a more generic follow up question was asked. A second interaction revealed a user disclosing escalating stressors and when CAR-E responded by asking how they might “connect with someone who supports them” the user replied, “I don’t have any support.” CAR-E responded with a brief expression of concern but then returned to a coaching prompt.

Finally, the most common feedback was frustration when the AI resisted offering advice, even when directly prompted by users. Many sought concrete suggestions to help advance their thinking, and the lack of response felt like a limitation. This surfaced when asking for ideas or recommendations or even when users explicitly attempted to get answers.

## Critical Reflection on your process

CAR-E demonstrates the potential of combining advanced AI technologies to facilitate meaningful coaching conversations that could complement human coaching relationships. Early development has prompted both specific technical refinement and broader insights into the opportunities and limitations of AI coaching conversations in medical education.

### Differentiating CAR-E from Generic LLM Chatbots: Design and Governance

Commercial LLMs (e.g., ChatGPT) provide broad conversational capabilities, but CAR-E was architected specifically for coaching conversations in medical education. Beyond prompt engineering or a specific underlying model, effective AI coaching employs system-level choices that address coaching objectives, safety requirements, and institutional governance. Generic LLMs default to solution-oriented responses. CAR-E is designed to anchor responses to evidence-based methods from our curated corpus ensuring adherence to coaching competencies such as reflective questioning, avoiding direct advice, and turning insight into action. CAR-E’s dual memory architecture enables conversational context to remember prior conversations, track goals, reference past breakthroughs, and recognizing patterns across sessions, these are capabilities generic LLMs cannot provide.

CAR-E runs in a FERPA-compliant Azure environment with Active Directory ensuring institutional governance with auditable access, role-based permissions, and retention controls, unlike commercial chat bots where data leave institutional boundaries. It also integrates speech-to-text and text-to-speech for real-time voice interaction, with turn-taking and latency tuned to support reflective pauses and concise prompts typical of coaching dialogue.

CAR-E’s safety inference layer is being built to detect any concerning disclosures (e.g. harmful behaviors, unhealthy coping) and trigger resource referral and escalation pathways, connecting users to human support when needed. The examples of users expressing red-flag disclosures illustrate the potential and current limitations of CAR-E’s approach to distress. Future iterations will incorporate some specific language for CAR-E to monitor and specific guidance on the importance of sustaining engagement in these critical moments rather than returning to a coaching mode.

Perhaps most critically, CAR-E represents responsible AI deployment in medical education given emerging evidence that LLMs may contribute to user disempowerment if tools are not intentionally designed to preserve learner agency and critical thinking [12]. CAR-E maintains explicit scope boundaries, (e.g., not therapy, not clinical advice) user notices, and monitors conversation attributes (e.g., balance of open questions, closure behaviors) for quality assurance. While CAR-E is intentionally designed to facilitate reflection rather than provide advice, explicit warnings and ongoing monitoring are necessary to ensure it does not give guidance that would conflict with institutional norms. Similar risk exists in human-led coaching, mentoring, or advising relationships. This system will ideally augment existing coaching resources, routing users to human support when conversations exceed the coaching remit, exemplifying responsible AI deployment in medical education.

### Evolving the Conversational Framework

A key lesson was that teaching CAR-E about coaching principles alone wasn’t enough, we also had to train it in conversational flow and structure. While human coaches intuitively navigate conversations using verbal and non-verbal cues, AI systems require more explicit structure. Future iterations of CAR-E will adopt a three-phase model based on International Coaching Federation competencies:

1. **Establishing focus** – defining the conversations purpose
2. **Exploring and evoking awareness** – deep questioning and reflection
3. **Moving toward insight and action** – synthesizing and planning next steps

This framework aims to provide sufficient structure to guide meaningful conversations while avoiding endless loops of questioning without resolution.

### Balancing Coaching Purity with AI coaching conversations

Strict adherence to non-directiveness, a coaching best practice, posed challenges. While CAR-E appropriately avoided giving advice, some users became frustrated when seeking ideas or guidance. Human coaches often use intuition to share timely insights or observations that spark new thinking. Training CAR-E has been challenging since giving advice is often a judgment call and not an absolute no. Future version may incorporate a hybrid approach: primarily reflective questioning, with occasional framing of suggestions or observations provided when users appear stuck and or explicitly ask for input. We continue to navigate this tension between users’ inclination for quick, directive answers and the richer, though more effortful experience of genuine coaching conversations. This tension is reflected in the coaching literature, which shows coaching improves performance, well-being, and attitudes but it is especially effective in improving the ability to regulate one’s own thinking and behavior rather than relying on directive advice [13].

### Exploring the Unique Position of AI Coaching

A coaching relationship is built on a longitudinal understanding of a coachee’s values and growth that extends beyond a single session. We are unable to know if users had authentic conversations or how they might interact with CAR-E if not asked, but the coaching conversations certainly appeared honest and vulnerable with complex emotions shared. This is promising for the possibility of AI coaching conversations as a complementary addition to human coaching relationships, even though it is still unclear how much trust and safety can be replicated with AI [14]. Regardless, given how well CAR-E resisted giving advice, it suggests AI-facilitated coaching conversations might align more faithfully with coaching competencies than those led by human coaches with minimal training [15]. In medical education, what is labeled as coaching often resembles mentoring or advising in practice, and AI coaching conversations may help better differentiate these roles [16]. One potential model is for human coaches to review CAR-E transcripts to accelerate and deepen their understanding of their users before subsequent conversations. Alternatively, AI coaching conversations may function to prime trainees, increasing their self-awareness and movement towards insight or action that can make in-person coaching more focused and productive. While CAR-E is designed for purposeful questioning to facilitate learner insight, monitoring is essential to ensure it augments rather than replaces meaningful human interactions with coaches, mentors, and supportive faculty. Future research will explore how AI and human coaching might work together to create a more comprehensive support system for medical trainees.

### Limitations and Next Steps

This project has limitations. Users were asked to stress test CAR-E with a relevant conversation and provide feedback. Thus, the topics discussed in coaching conversations might change if they were prompted differently. In addition, the lessons learned from user’s feedback with CAR-E were organized for ease of understanding, but the content was based primarily on open-ended prompts, and a different structure may have produced other insights. Next steps include evaluating CAR-E with defined learner cohorts beyond a convenience sample and exploring structured curricular integration to better understand its use, memory, and impact. Future studies may also employ standardized or comparative designs to examine how AI-facilitated coaching conversations can augment, rather than replace human coaching.

## Conclusion

Coaching in medical education is often constrained by time, cost, perceived judgment, and power dynamics. By addressing the opportunities and challenges identified in this innovation, we are working to refine AI coach conversations that expand access to reflective, growth-oriented dialogue, while honoring the indispensable role of human connection in medical education.
